# Carotenoid Profiling of a Red Seaweed *Pyropia yezoensis*: Insights into Biosynthetic Pathways in the Order Bangiales

**DOI:** 10.3390/md16110426

**Published:** 2018-11-01

**Authors:** Jiro Koizumi, Naoki Takatani, Noritoki Kobayashi, Koji Mikami, Kazuo Miyashita, Yumiko Yamano, Akimori Wada, Takashi Maoka, Masashi Hosokawa

**Affiliations:** 1 Graduate School of Fisheries Sciences, Hokkaido University, Hakodate 041-8611, Japan; j_koizumi@eis.hokudai.ac.jp (J.K.); n-takatani@eis.hokudai.ac.jp (N.T.); air-norsquare.el-bor@ezweb.ne.jp (N.K.); 2 Faculty of Fisheries Sciences, Hokkaido University, Hakodate 041-8611, Japan; komikami@fish.hokudai.ac.jp (K.M.); kmiya@fish.hokudai.ac.jp (K.M.); 3 Laboratory of Organic Chemistry for Life Science, Kobe Pharmaceutical University, Kobe 658-8558, Japan; y-yamano@kobepharma-u.ac.jp (Y.Y.); a-wada@kobepharma-u.ac.jp (A.W.); 4 Research Institute for Production Development, 15 Shimogamo, Morimoto Cho, Sakyoku, Kyoto 606-0805, Japan; maoka@mbox.kyoto-inet.or.jp

**Keywords:** *Pyropia yezoensis*, α-cryptoxanthin, zeinoxanthin, lutein-5,6-epoxide, antheraxanthin, carotenoid synthesis pathway, red seaweed

## Abstract

Carotenoids are natural pigments that contribute to light harvesting and photo-protection in photosynthetic organisms. In this study, we analyzed the carotenoid profiles, including mono-hydroxy and epoxy-carotenoids, in the economically valuable red seaweed *Pyropia yezoensis*, to clarify the detailed biosynthetic and metabolic pathways in the order Bangiales. *P. yezoensis* contained lutein, zeaxanthin, α-carotene, and β-carotene, as major carotenoids in both the thallus and conchocelis stages. Monohydroxy intermediate carotenoids for the synthesis of lutein with an ε-ring from α-carotene, α-cryptoxanthin (β,ε-caroten-3’-ol), and zeinoxanthin (β,ε-caroten-3-ol) were identified. In addition, β-cryptoxanthin, an intermediate in zeaxanthin synthesis from β-carotene, was also detected. We also identified lutein-5,6-epoxide and antheraxanthin, which are metabolic products of epoxy conversion from lutein and zeaxanthin, respectively, by LC-MS and ^1^H-NMR. This is the first report of monohydroxy-carotenoids with an ε-ring and 5,6-epoxy-carotenoids in Bangiales. These results provide new insights into the biosynthetic and metabolic pathways of carotenoids in red seaweeds.

## 1. Introduction

Carotenoids are yellow-orange tetraterpenoid pigments generated from C4 isoprenoid units. To date, more than 700 carotenoids have been identified in nature. Carotenoids are synthesized in plants, algae (unicellular type), seaweeds (macrophytic type), bacteria, and fungi, but not animals. In photosynthetic organisms, carotenoids contribute to photosynthesis in light-harvesting antenna complexes in the thylakoid membrane of chloroplasts. They also play an important role in photo-protection from damage by reactive oxygen species under excess light energy [1]. In addition, carotenoids have various health benefits in humans [2]. For example, β-carotene, α-carotene, and β-cryptoxanthin have pro-vitamin A activity [3]. Lutein and zeaxanthin decrease the risk of age-related macular degeneration [4,5].

*Pyropia yezoensis*, belonging to Bangiales (Rhodophyta), is one of the most economically valuable marine foods in East Asia. In Japan, approximately 300,000–350,000 tons (wet weight) of thalli are produced every year [6]. Moreover, *P. yezoensis* is valuable for studies of seaweed biology owing to its heteromorphic life cycle, characterized by independent macroscopic leafy gametophyte (thallus) and microscopic, filamentous sporophyte (conchocelis) stages [7]. The genome sequence of *P. yezoensis* has been determined [8] and it is readily cultivated at a laboratory scale; accordingly, it is a useful model organism for studies of red seaweeds [9].

**Figure 1:**
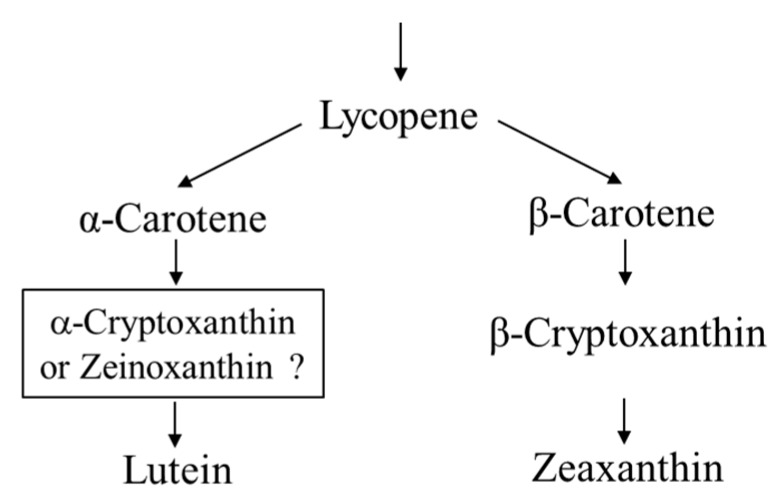
Occurrence of carotenoids in *Pyropia yezoensis*.

In most red seaweeds, major carotenoids include α-carotene, β-carotene, lutein, and zeaxanthin [10,11,12]. Lutein consists of two hydroxyl groups with α-carotene composed of a β-ring and ε-ring, and thus, has an asymmetrical structure. In terrestrial plants, lutein is produced by two biosynthetic pathways via α-cryptoxanthin (β,ε-caroten-3’-ol) and zeinoxanthin (β,ε-caroten-3-ol) from α-carotene by carotene hydroxylases, such as CYP97A, CYP97C, and BHY [13]. In the genome of *Porphyra umbilicalis*, which belongs to Bangiales, *CYP97A*, *CYP97C*, and *BHY* genes have not been found [14]. PuCHY_1_ belonging to the CYP97B subfamily shows β-hydroxylation activity in the synthesis of zeaxanthin from β-carotene in *Po. umbilicalis* [15]. Moreover, intermediate monohydroxy carotenoids in lutein synthesis from α-carotene with an ε-ring are not well known (Figure 1). These results suggest that Bangiales have characteristic biosynthetic pathways different from those of land plants. Therefore, it is interesting to investigate the lutein synthesis pathway in *P. yezoensis*, including intermediate carotenoids.

We recently found a gene candidate for zeaxanthin epoxidase (ZEP), which catalyzes the conversion from zeaxanthin to violaxanthin, in *P. yezoensis* [16]. Abscisic acid (ABA), a phytohormone, has also been detected in *P. yezoensis* [17]. Genes involved in ABA biosynthesis have also been found in the genomes of Bangiales. ABA attenuates oxidative stress under desiccation in seaweeds [18]. These findings suggest that intermediates in the biosynthetic pathway of ABA from zeaxanthin might be found in Bangiales. However, owing to the lack of information about epoxy-carotenoids, their identification is necessary to clarify the ABA synthetic pathway in this order.

In the present study, we analyzed intermediate carotenoids to clarify the carotenoid synthetic pathways in *P. yezoensis*. Based on the identification of monohydroxy-carotenoids and epoxy-carotenoids, we proposed novel carotenoid metabolic pathways in *P. yezoensis*.

## 2. Results

### 2.1. Lutein, Zeaxanthin, and α/β-Carotene Contents in P. yezoensis

**Figure 2:**
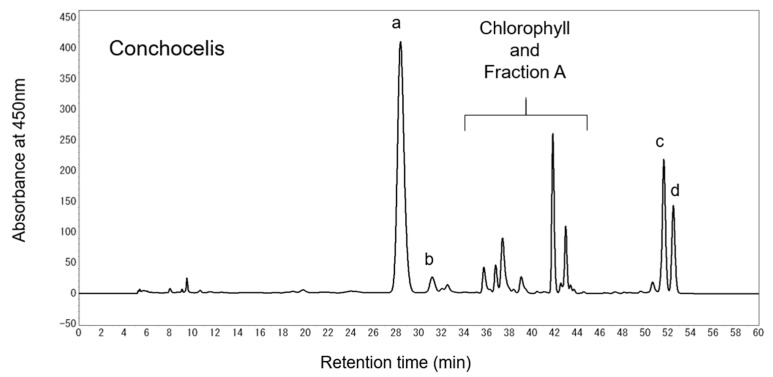
Chromatogram of total lipids extracted from the conchocelis of *Pyropia yezoensis*. Carotenoids were measured using a high-performance liquid chromatography (HPLC) system equipped with a diode array detector at 450 nm. Two Develosil C30-UG-5 columns were connected for the analysis. The mobile phase was methanol for 20 min, followed by dichloromethane, increased linearly from 0% to 50% over 20 min, and methanol: Dichloromethane (1:1, *v*/*v*) for an additional 20 min. The flow rate was 1.0 mL/min, and the sample injection volume was 50 μL. a, lutein; b, zeaxanthin; c, α-carotene; d, β-carotene.

**Figure 3:**
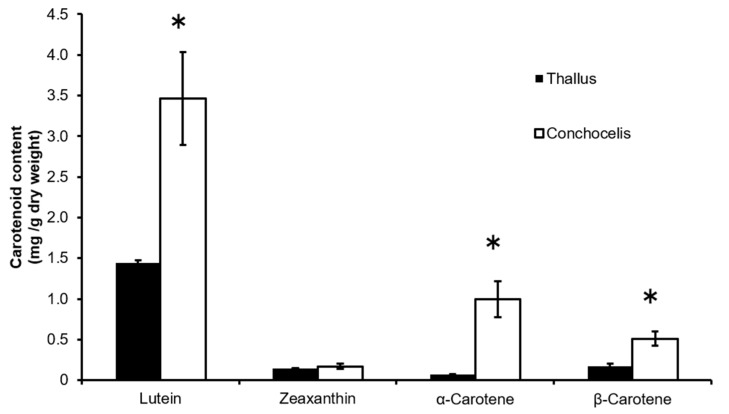
Carotenoid contents in the thallus and conchocelis of *Pyropia yezoensis*. Values are the means ± SD (*n* = 3). Asterisks show significance by the Student’s *t*-test (* *p* < 0.05).

Total lipids were extracted from *P. yezoensis* at the conchocelis stage, and major carotenoids were analyzed by high-performance liquid chromatography (HPLC) at 450 nm (Figure 2). Carotenoids were identified by comparing the HPLC retention times and absorption spectra with those of their authentic standards. Lutein, zeaxanthin, α-carotene, and β-carotene were detected as major carotenoids in the total lipids from *P. yezoensis*. Lutein was the most abundant carotenoid at 3.46 ± 0.57 mg/g dry weight and 1.44 ± 0.03 mg/g dry weight in the cultured conchocelis and the aquacultured thallus of *P. yezoensis*, respectively. The contents of these carotenoids were greater in the conchocelis than in the thallus of *P. yezoensis* (Figure 3).

### 2.2. Isolation and Identification of Monohydroxy-Carotenoids in P. yezoensis

**Figure 4:**
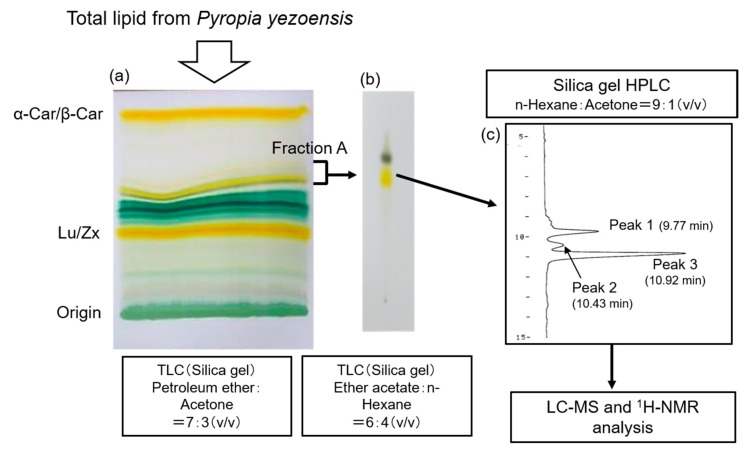
Scheme for the separation of monohydroxyl carotenoids in total lipids extracted from the conchocelis of *Pyropia yezoensis*. (**a**) Silica gel TLC of total lipids extracted from the conchocelis of *P. yezoensis*; (**b**) Silica gel TLC of Fraction A to remove the chlorophyll fraction; (**c**) Fractionation of peak 1, peak 2, and peak 3 in Fraction A by an HPLC system equipped with a Mightysil silica gel column (250 × 4.6 mm), with a flow rate of 1.0 mL/min with n-hexane: acetone (9:1, *v*/*v*) and detection at 450 nm. α-Car/β-Car: α-carotene and β-carotene; Lu/Zx: lutein and zeaxanthin.

We observed a yellow band (Fraction A in Figure 4a) between α/β-carotene and lutein/zeaxanthin on the silica gel by thin layer chromatography (TLC) using total lipids from the *P. yezoensis* conchocelis. To remove chlorophyll in Fraction A eluted from the silica gel with acetone, silica gel TLC was performed using ethyl acetate: n-hexane (6:4, *v*/*v*) (Figure 4b). Peak 1, peak 2, and peak 3 were separated from Fraction A by silica gel-HPLC with n-hexane: Acetone (9:1, *v*/*v*) (Figure 4c).

**Figure 5:**
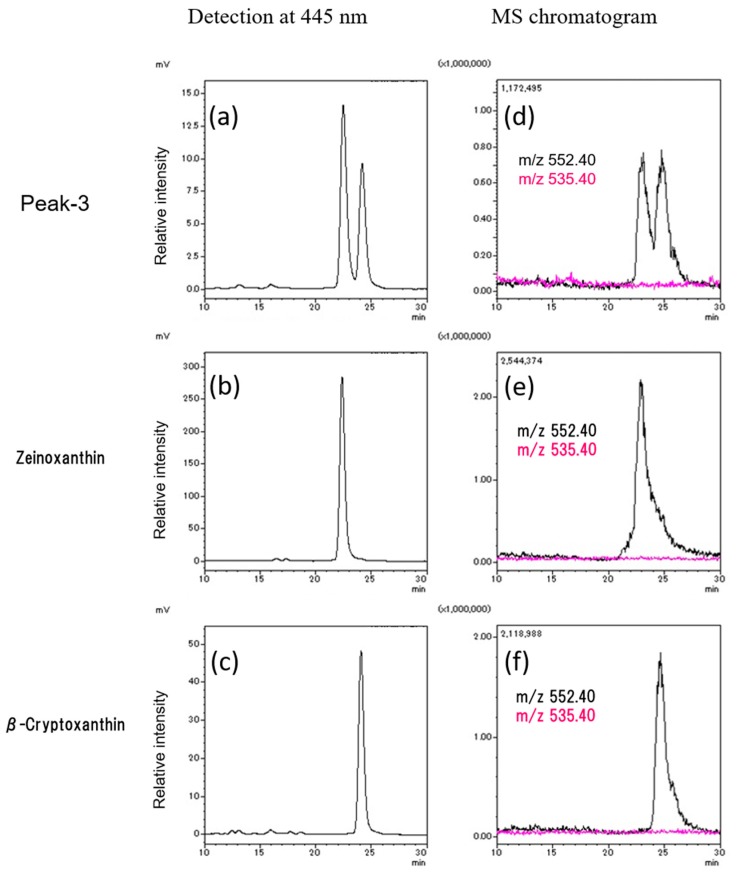
LC-MS analysis of peak 3 separated from the conchocelis of *Pyropia yezoensis*. Peak-3 separated from Fraction A was evaluated by LC-MS (Shimadzu LCMS8040) with an ODS-UG-3 column and methanol as a solvent at 0.1 mL/min. Detection at 445 nm of (**a**) peak 3 separated from the conchocelis of *Pyropia yezoensis*, (**b**) zeinoxanthin standard, (**c**) β-cryptoxanthin standard. MS chromatogram at *m/z* 552.40 (black) and *m/z* 535.40 (red) of (**d**) peak 3 separated from the conchocelis of *Pyropia yezoensis*, (**e**) zeinoxanthin standard, (**f**) β-cryptoxanthin standard.

Peak 3 was further separated into peak 3-1 and peak 3-2 using the LC-MS system (Shimadzu Triple Quadrupole Mass Spectrometer LCMS8040) with an ODS column and methanol as a solvent (Figure 5a). The retention times of peak 3-1 and peak 3-2 detected at 445 nm were the same as those of the zeinoxanthin and β-cryptoxanthin standards, respectively (Figure 5a–c). Mass chromatography at *m/z* 552.40 [M]^+^ also indicated the same patterns as those of the zeinoxanthin and β-cryptoxanthin standards, respectively (Figure 5d–f)). Furthermore, product ions at *m/z* 535.40 [MH-H_2_O]^+^ were not detected in peak 3-1 and peak 3-2, consistent with zeinoxanthin and β-cryptoxanthin with an OH group on the β-ring, but not on the ε-ring. These data indicated that peak 3-1 and peak 3-2 were zeinoxanthin and β-cryptoxanthin, respectively.

**Figure 6:**
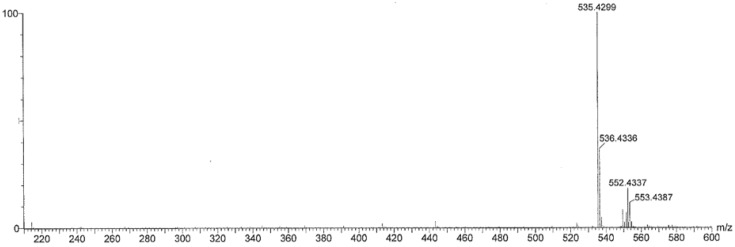
Mass spectrum of peak 1 separated from the conchocelis of *Pyropia yezoensis*.

Peak 1 (Figure 4c) corresponded to a minor carotenoid compared to peak 3, which included zeinoxanthin and β-cryptoxanthin. Therefore, we analyzed the peak by TOF-MS (Waters Acquity LC Xevo G2-S Q TOF Mass Spectrometer) with high sensitivity. An LC-MS analysis of peak 1 showed ions at *m/z* 552.4337 [M]^+^ (C_40_H_56_O, calc. for 552.4331) and *m/z* 535.4299 [MH-H_2_O]^+^ as products (Figure 6). UV-VIS wavelengths were 420, 444, and 471 nm (methanol, Table 1). In particular, [MH-H_2_O]^+^ product ions showed higher intensities than those of [M]^+^ molecular ions. OH group binding at the 3′ position of the ε-ring more easily produces –H_2_O product ions compared with binding to the hydroxylated β-ring in zeinoxanthin and β-cryptoxanthin. Furthermore, ^1^H-NMR signals indicate that peak 1 is α-cryptoxanthin with an OH group at the allylic position on the ε-end group [19] (Table 1).

We tried to identify peak 2 in the total lipids isolated from *P. yezoensis*. However, it was difficult to determine the structure owing to the low content of the compound.

These results showed that *P. yezoensis* contains three monohydroxy-carotenoids, i.e., α-cryptoxanthin, β-cryptoxanthin, and zeinoxanthin, as intermediate carotenoids in the biosynthetic pathways from α-carotene to lutein or β-carotene to zeaxanthin.

### 2.3. Isolation and Identification of Epoxy-Carotenoids in P. yezoensis

**Figure 7:**
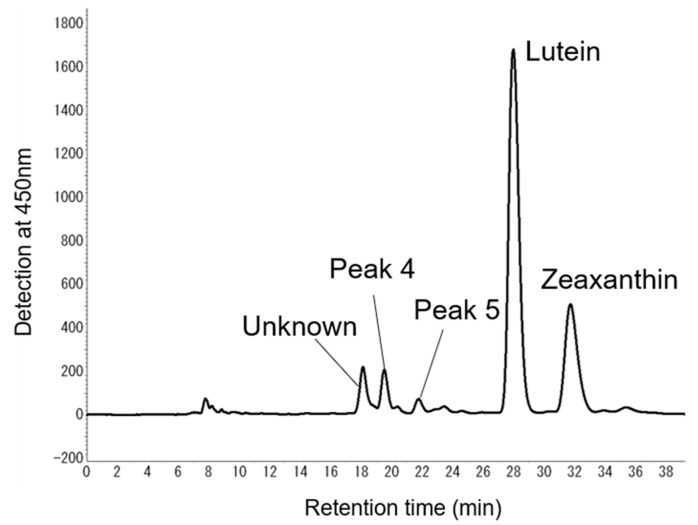
Purification of peak 4 and peak 5 in Fraction B separated from the thallus of *Pyropia yezoensis* by the HPLC system. Peak 4 and Peak 5 were isolated by the HPLC system equipped with a diode array detector at 450 nm. Two Develosil C30-UG-5 columns were connected and used for carotenoid isolation. The mobile phase was methanol. The flow rate was 1.0 mL/min.

We evaluated epoxy-carotenoids to reveal novel carotenoid metabolic pathways in *P. yezoensis*. We extracted total lipids from the aquacultured thallus of *P. yezoensis*, and the concentrated polar carotenoid fraction (Fraction B) was expected to contain epoxy-carotenoids according to silica gel open column chromatography. Furthermore, by HPLC separation using a C30 column (Develosil C30-UG-5, two columns were connected to enhance separation), peak 4 and peak 5 were isolated from Fraction B (Figure 7).

**Figure 8:**
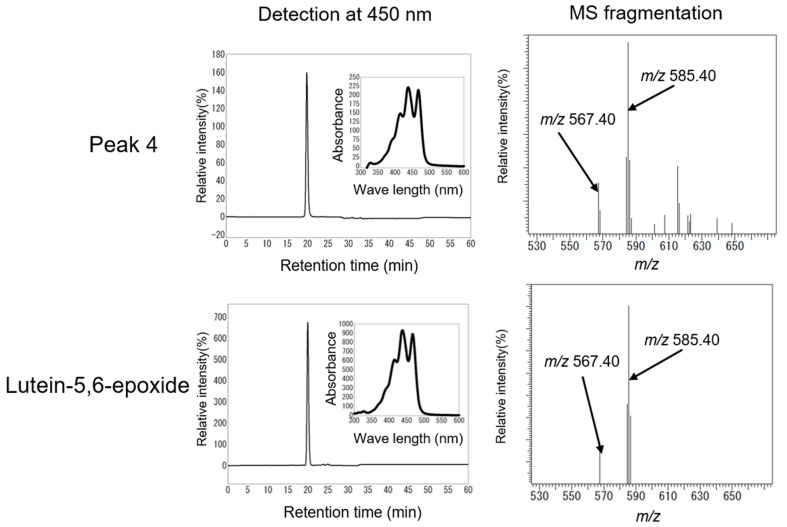
HPLC and LC-MS analyses of peak 4 and lutein-5,6-epoxide.

**Figure 9:**
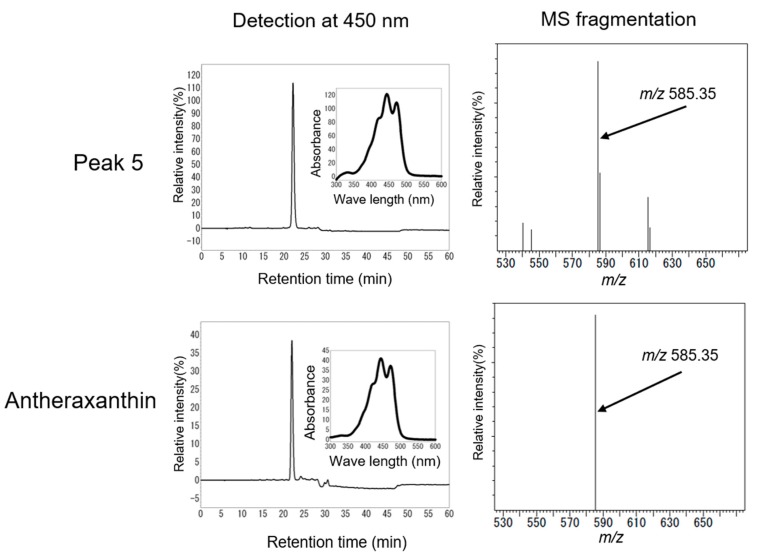
HPLC and LC-MS analyses of peak 5 and antheraxanthin.

Peak 4 had the same HPLC retention time (19.80 min) and maximal absorption wavelength as those of the lutein-5,6-epoxide standard (Figure 8). An LC-MS (Shimadzu LCMS8040) analysis of peak 4 showed that the [MH]^+^ ion and product ion [MH-18]^+^ at *m/z* 585.40 and *m/z* 567.40 were identical to those of the standard. ^1^H-NMR data indicated that peak 4 is lutein-5,6-epoxide (Table 2). The HPLC retention time (22.18 min), maximal absorption wavelength, LC-MS, and ^1^H-NMR data showed that peak 5 is antheraxanthin (5,6epoxide derived from zeaxanthin) (Figure 9, Table 2). This is the first identification of lutein-5,6-epoxide and antheraxanthin in Bangiales.

## 3. Discussion

Bangiales contains not only β-carotene and its derivatives, but also α-carotene and lutein with an ε-ring [11,15], and these carotenoids have important biological and nutraceutical functions in red seaweeds [20,21]. In the present study, we analyzed the carotenoid profile of one of the most popular edible red seaweeds, *P. yezoensis*.

Lutein was the predominant carotenoid in the conchocelis and thallus of *P. yezoensis*, similar to other red seaweeds (Figure 3). Lutein and zeaxanthin have established health benefits, such as protection against aged-related macular degeneration [4,5]. In addition, the improvement of cognitive function by dietary lutein and zeaxanthin has been reported in a clinical trial [22]. Thus, *P. yezoensis* containing lutein and zeaxanthin is a highly valuable food with respect to human health.

**Figure 10:**
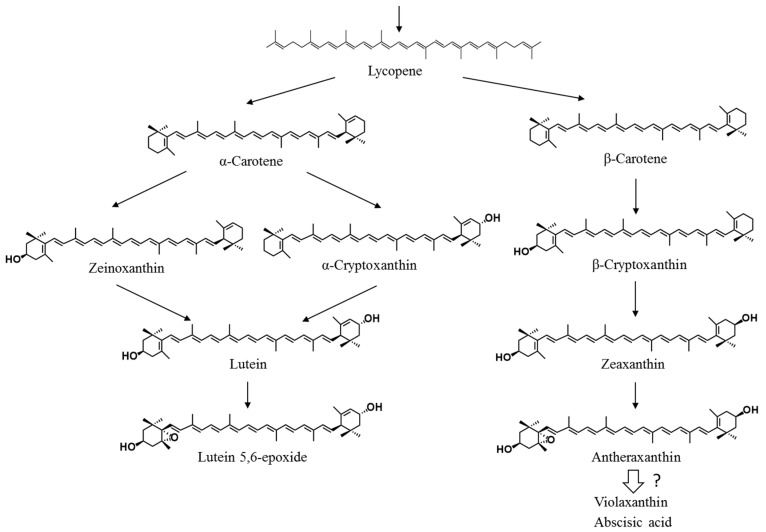
Summary of proposed pathways of carotenoid synthesis and metabolism in *Pyropia yezoensis*.

Minor intermediate carotenoids, which are important components of carotenoid biosynthetic pathways, have not been comprehensively identified [11,12]. We identified intermediate carotenoids in *P. yezoensis* and found that both α-cryptoxanthin and zeinoxanthin, which are monohydroxy-carotenoids, are produced as intermediates in the synthesis of lutein from α-carotene in *P. yezoensis* (Figure 10). α-Cryptoxanthin has been detected in the red seaweeds *Antithamnion plumula* (Ceramiales) and *Jania rubens* (Corallinales) [23,24]. However, α-cryptoxanthin has not been detected in Bangiales to date. To the best of our knowledge, this is the first report of both zeinoxanthin and α-cryptoxanthin in *P. yezoensis* of Bangiales, as evidenced by LC-MS and ^1^H-NMR analyses.

In higher plants, lutein is synthesized via α-cryptoxanthin and zeinoxanthin from α-carotene [25]. The hydroxylation of α-carotene is catalyzed by nonheme diiron β-hydroxylase (BHY) and heme-containing cytochrome P450-type carotene hydroxylase (CYP97A and C) [26,27,28]. CYP97C1 (the *Lut1* locus) catalyzes the ε-ring hydroxylation of both α-carotene and zeinoxanthin [28], and CYP97A3 catalyzes β-ring hydroxylation in *Arabidopsis thaliana* [13]. In the liverwort *Marchantia polymorpha*, CYP97C catalyzes the ε-ring hydroxylation of zeinoxanthin but not α-carotene, and BHY catalyzes β-ring hydroxylation [29]. The differences in lutein biosynthesis among taxa are likely to be associated with differences in the specificity of carotene hydroxylases for β- and ε-rings in the α-carotene molecules.

Recently, PuCHY1, which belongs to the CYP97B subfamily, was functionally characterized as a carotene hydroxylase in zeaxanthin synthesis from β-carotene in *Po. umbilicalis* [15]. It is noteworthy that there are no homologues of *CYP97A*, *CYP97C*, and *BHY* genes in the genomes of *Po. umbilicalis* [14] and *P. yezoensis* [unpublished observation]. Therefore, the identification of α-cryptoxanthin and zeinoxanthin together with genome information provides insight into the lutein biosynthetic pathway with unique enzymes in Bangiales.

Antheraxanthin and violaxanthin are converted from zeaxanthin in land plants [30]. The reversible conversion among these carotenoids in response to light stress plays a pivotal role in photoprotection and photoabsorption, and is referred to as the xanthophyll cycle [31]. The xanthophyll cycle requires two enzymes, i.e., zeaxanthin epoxidase (ZEP), which converts zeaxanthin to violaxanthin via antheraxanthin, and violaxanthin de-epoxidase, which converts violaxanthin to zeaxanthin [30]. Antheraxanthin has been detected in red seaweeds in the orders Corallinales, Ceramiales, and Gracilariales, within the class Florideophyceae [11]. However, it has not been detected in Bangiales [11]. In the present study, we evaluated epoxy-carotenoids from zeaxanthin in *P. yezoensis*, because we previously found a ZEP-related sequence in the *P. yezoensis* genome, as well as ABA, which is a down-stream product of violaxanthin [17]. Since it was difficult to detect epoxy-carotenoids directly in total lipids extracted from *P. yezoensis*, the polar lipid fraction (Fraction B) containing epoxy-carotenoids was first separated from the total lipids by silica gel open column chromatography. Then, peak 4 and peak 5 (Figure 7) were isolated from the polar lipid fraction by HPLC and their structures were determined by LC-MS and ^1^H-NMR analyses. The analytical data demonstrated that peak 4 and peak 5 in *P. yezoensis* lipids were lutein-5,6-epoxide and antheraxanthin with an epoxide group, respectively. These results provide the first evidence of these 5,6-epoxy carotenoids in *P. yezoensis*, belonging to Bangiales, and provide insight into the epoxidation pathways for lutein and zeaxanthin (Figure 10), although its activity is weak as indicated by the low contents of epoxy-carotenoids.

A ZEP-related sequence has been detected in the genomes of Bangiales [17]. Dautermann and Lohr [32] reported ZEP enzyme activity in *Madagascaria erythrocladioides* (order Erythropeltidales), and the accumulation of antheraxanthin as a predominant carotenoid in a ZEP-deficient tobacco mutant. A distant relationship between the ZEP protein candidates from *P. yezoensis* and *M. erythrocladioides* was observed in an in silico analysis. In addition, the ZEP protein candidate in *P. yezoensis* is not involved in carotenoid metabolism based on its lack of a transit peptide and the lack of epoxy-carotenoids [32]. However, in the present study, we clearly detected antheraxanthin and lutein-5,6-epoxide in *P. yezoensis* (Figure 8 and Figure 9). Since antheraxanthin and lutein-5,6-epoxide are derived from zeaxanthin and lutein by ZEP, our findings provide evidence for ZEP activity in *P. yezoensis*. Moreover, the production of antheraxanthin may be related to violaxanthin synthesis in *P. yezoensis* (Figure 10). The present results contribute to the elucidation of the biosynthesis of epoxy-carotenoids in the order Bangiales.

## 4. Materials and Methods

### 4.1. Chemicals

α-Carotene, β-carotene, β-cryptoxanthin, lutein, zeaxanthin, antheraxanthin, and lutein-5,6-epoxide were purchased from Carote *Nature* GmbH (Münsingen, Switzerland). Zeinoxanthin used for standard was synthesized by Wittig condensation of C_25_-apocarotenal derived from commercially available α-ionone, with previously prepared C_15_-phosphonium salt possessing 3-hyxroxy-β-end group [33]. ^1^H NMR data of synthetic zeinoxanthin were identical with those reported in Reference [34].

### 4.2. Seaweed Materials

Thalli and conchocelis filaments of *P. yezoensis* strain U-51 were used in the present study. Thalli maricultured at Shichigahama (Miyagi, Japan) were frozen and kept under −20 °C until extraction of total lipid. Conchocelis materials were prepared by laboratory culture in ESL medium [35] at 15 °C, under irradiation of 40 μmol photons m^−2^ s^−1^ provided by cool white fluorescent lamps with a photoperiod of 10 h light/14 h dark. The medium was bubbled continuously with filter-sterilized air and changed weekly. We used approximately 40–80 mg of conchocelis stage of *P. yezoensis* during cultivation for total lipid extraction.

### 4.3. Extraction of Total Lipids from P. yezoensis

Total lipid was extracted from conchocelis and thallus of *P. yezoensis* with 20-fold methanol (*v*/*w*) for 24 h at room temperature under shading conditions. The extraction with methanol was conducted twice. Then, methanol was evaporated, and the extracts was solved in chloroform-methanol-water (10:5:3, *v*/*v*/*v*) to remove water soluble components. The total lipid fraction containing carotenoids was obtained from chloroform layer.

### 4.4. Lutein, Zeaxanthin, α/β-Carotene Contents in P. yezoensis

Carotenoids content in *P. yezoensis* were measured by a high-performance liquid chromatography (HPLC) system (Hitachi, Tokyo, Japan) equipped with a diode array detector (Hitachi L2455, Tokyo, Japan). Two Develosil C30-UG-5 (Nomura Chemical Co., Aichi, Japan) columns were connected and used for carotenoid analysis. Detection was set at 450 nm. Mobile phase was methanol until 20 min, and thereafter, dichloromethane content increased linearly from 0% to 50% in 20 min, then was held at methanol:dichloromethane (1:1, *v*/*v*) for additional 20 min. The flow rate was maintained at 1.0 mL/min, and sample injection volume was 50 μL. α-Carotene, β-carotene, lutein, and zeaxanthin were identified by comparison of retention time and absorption spectra of HPLC analysis with their authentic standards. The contents of α-carotene, β-carotene, lutein, and zeaxanthin in *P. yezoensis* were calculated using a calibration curve prepared by each authentic standard.

### 4.5. Isolation of Monohydroxy Carotenoids

The total lipid of *P. yezoensis* was developed on a silica gel TLC plate (Merk KGaA, Darnstadt, Germany) with petroleum ether:acetone (7:3, *v*/*v*) or n-hexane:acetone (7:3, *v*/*v*). Three pigment fractions with yellow-orange color were observed on the TLC plate. The yellow pigment fraction with relative front (Rf) value 0.61 between strong two yellow-orange fractions with Rf value 0.92 (α/β-carotene) and 0.42 (lutein/zeaxanthin) was scrapped off from the TLC plate developed with petroleum ether:acetone (7:3, *v*/*v*), and extracted with acetone. Separation of the yellow pigment fraction (Fraction A) was conducted by silica gel TLC plate with ethyl acetate:n-hexane (6:4, *v*/*v*) to remove remaining chlorophyll. Then, peak 1, peak 2, and peak 3 were separated from Fraction A by HPLC system (Hitachi, Tokyo, Japan) equipped with Mightysil silica gel column (Kanto Chemical Co., Tokyo, Japan, 250 × 4.6 mm), flow rate: 1.0 mL/min with n-hexane:acetone (9:1, *v*/*v*), detection at 450 nm. Peak 3 was further fractionated and analyzed using an LC-MS system (Shimadzu Triple Quadrupole mass spectrometer LCMS8040, Shimadzu, Kyoto, Japan), equipped with ODS column (Develosil ODS-UG-3 150 × 2.0 mm, Nomura Chemical Co., Aichi, Japan). Methanol was used as a mobile phase at a flow rate of 0.1 mL/min.

### 4.6. Isolation of Epoxy-Carotenoids

The total lipid extracted from aquacultured thallus of *P. yezoensis* was separated silica gel column by n-hexane:acetone (7:3, *v*/*v*). Four fractions [strong yellow layer (α/β-carotene fraction), yellow-green layer (α/β-cryptoxanthin, zeinoxanthin and chrolophyll faction), strong orange layer (lutein/zeaxanthin fraction), and light-yellow layer (Fraction B)] were eluted in order. Then, peak 4 and peak 5 were isolated from Fraction B (light yellow layer) using an HPLC system (Hitachi, Tokyo, Japan) equipped with a Develosil C30-UG-5 column (250 × 4.6 mm, two columns were connected), flow rate: 1.0 mL/min with methanol, detection at 450 nm.

### 4.7. Identification of Monohydroxy- and Epoxy-Carotenoids in P. yezoensis

The chemical structures of peaks 1–5 were determined by LC-MS and ^1^H-NMR analyses. LC-MS analysis was carried out using a Shimadzu Triple Quadrupole mass spectrometer LCMS8040, as described in 2.6 or a Waters Xevo G2S Q TOF mass spectrometer (Waters Corporation, Milford, CT, USA), equipped with an Acquity UPLC system with Acquity 1.7 μm BEH UPLC C18 column (100 × 2.1 mm) (Waters Corporation, Milford, CT, USA), and methanol as a mobile phase at a flow rate of 0.4 mL/min. ^1^H-NMR (500 MHz) spectra in CDCl_3_ were measured with an UNITY INOVA-500 system (Varian Corporation, Palo Alto, CA, USA).

### 4.8. Statistical Analysis

Analytical data of carotenoid content (Figure 3) were expressed as the mean ± standard deviation (SD). Statistical significance was determined between the two groups using the Student’s *t*-test. A significant difference was defined at *p* < 0.05.

## 5. Conclusions

The results of the present study demonstrated that *P. yezoensis* synthesizes zeinoxanthin and α-cryptoxanthin, as well as β-cryptoxanthin, as intermediate monohydroxy-carotenoids. These results indicate that *P. yezoensis* has two lutein biosynthetic pathways via zeinoxanthin and α-cryptoxanthin from α-carotene (Figure 10). Furthermore, antheraxanthin and lutein-5,6-epoxide were found for the first time in Bangiales (Figure 10). In particular, antheraxanthin is considered an intermediate carotenoid in the conversion from zeaxanthin to violaxanthin. The carotenoid profile of *P. yezoensis* provides new insight into the biosynthetic and metabolic pathways of carotenoids in red seaweeds.

## Figures and Tables

**Table 1:** Spectrum data of peak 1 isolated from the conchocelis of *Pyropia yezoensis*.

UV-VIS	420, 444, 471 nm Methanol
^1^H-NMR	δ ppm
16/17	1.03
18	1.73
19	1.97
20	1.97
4’	5.55
7’	5.43
17’	0.85
18’	1.63
19’	1.91
20’	1.97

**Table 2:** ^1^H-NMR spectral data of peak 4 and peak 5 isolated from the thallus of *Pyropia yezoensis*.

	Peak 4		Peak 5	
Position	d	mult. J (Hz)	d	mult. J (Hz)
2	1.25	dd (12, 7)	1.25	dd (12, 7)
2	1.63	ddd (12, 3, 1.5)	1.63	ddd (12, 3, 1.5)
3	3.91	m	3.91	m
4	1.63	dd (14, 9)	1.63	dd (14, 9)
4	2.39	ddd (14, 5, 1.5)	2.39	ddd (14, 5, 1.5)
7	5.88	d (16)	5.88	d (16)
8	6.29	d (16)	6.29	d (16)
10	6.20	d (11)	6.20	d (11)
11	6.61	dd (15, 11)	6.61	dd (15, 11)
12	6.38	d (15)	6.38	d (15)
14	6.25	m	6.25	m
15	6.63	m	6.63	m
16	0.98	s	0.98	s
17	1.15	s	1.15	s
18	1.19	s	1.19	s
19	1.93	s	1.93	s
20	1.97	s	1.97	s
2’	1.37	dd (13, 7)	1.48	
2′	1.85	dd (13, 6)	1.77	
3′	4.25	m	4.00	m
4′	5.55	br. S	2.05	dd (14, 9)
4′			2.39	ddd (14, 5.5, 1.5)
6′	2.40	d (9)		
7′	5.43	dd (15.5, 9)	6.10	d (16)
8′	6.14	d (16)	6.16	d (16)
10′	6.14	d (10)	6.16	d (11)
11′	6.62	dd (15, 11)	6.60	dd (15, 11)
12′	6.36	d (15)	6.35	d (15)
14′	6.26	m	6.25	d (11)
15′	6.63	m	6.63	m
16′	1.00	s	1.08	s
17′	0.85	s	1.08	s
18′	1.63	s	1.74	s
19′	1.91	s	1.93	s
20′	1.97	s	1.97	s
